# Editor’s Note: Adult bone marrow mesenchymal and neural crest stem cells are chemoattractive and accelerate motor recovery in a mouse model of spinal cord injury

**DOI:** 10.1186/s13287-021-02197-w

**Published:** 2021-02-15

**Authors:** Virginie Neirinckx, Gulistan Agirman, Cécile Coste, Alice Marquet, Valérie Dion, Bernard Rogister, Rachelle Franzen, Sabine Wislet

**Affiliations:** 1 Groupe Interdisciplinaire de Génoprotéomique Appliquée (GIGA),Neurosciences Research Center, Unit of Nervous system disorders andtreatment, University of Liège, Tour de Pathologie 2, Avenue de l’Hôpital, 1,4000 Liège, Belgium; 2 GIGA, Development, Stem Cells and RegenerativeMedicine Research Center, University of Liège, Liège, Belgium; 3 NeurologyDepartment, University Hospital, Liège, Belgium

**Editor’s Note to: Stem Cell Res Ther 6, 211 (2015)**

**https://doi.org/10.1186/s13287-015-0202-2**

**Editor’s Note**

Readers are alerted that concerns have been raised regarding Figures 1A and S1 [1]. We will update readers once we have further information and all parties have been given an opportunity to respond in full.
